# Effect of alendronate on post-traumatic osteoarthritis induced by anterior cruciate ligament rupture in mice

**DOI:** 10.1186/s13075-015-0546-0

**Published:** 2015-02-16

**Authors:** Mohammad S Khorasani, Sindi Diko, Allison W Hsia, Matthew J Anderson, Damian C Genetos, Dominik R Haudenschild, Blaine A Christiansen

**Affiliations:** Department of Orthopaedic Surgery, University of California-Davis Medical Center, 4635 2nd Ave, Suite 2000, Sacramento, CA 95817 USA

## Abstract

**Introduction:**

Previous studies in animal models of osteoarthritis suggest that alendronate (ALN) has antiresorptive and chondroprotective effects, and can reduce osteophyte formation. However, these studies used non-physiologic injury methods, and did not investigate early time points during which bone is rapidly remodeled prior to cartilage degeneration. The current study utilized a non-invasive model of knee injury in mice to investigate the effect of ALN treatment on subchondral bone changes, articular cartilage degeneration, and osteophyte formation following injury.

**Methods:**

Non-invasive knee injury via tibial compression overload or sham injury was performed on a total of 90 mice. Mice were treated with twice weekly subcutaneous injections of low-dose ALN (40 μg/kg/dose), high-dose ALN (1,000 μg/kg/dose), or vehicle, starting immediately after injury until sacrifice at 7, 14 or 56 days. Trabecular bone of the femoral epiphysis, subchondral cortical bone, and osteophyte volume were quantified using micro-computed tomography (μCT). Whole-joint histology was performed at all time points to analyze articular cartilage and joint degeneration. Blood was collected at sacrifice, and serum was analyzed for biomarkers of bone formation and resorption.

**Results:**

μCT analysis revealed significant loss of trabecular bone from the femoral epiphysis 7 and 14 days post-injury, which was effectively prevented by high-dose ALN treatment. High-dose ALN treatment was also able to reduce subchondral bone thickening 56 days post-injury, and was able to partially preserve articular cartilage 14 days post-injury. However, ALN treatment was not able to reduce osteophyte formation at 56 days post-injury, nor was it able to prevent articular cartilage and joint degeneration at this time point. Analysis of serum biomarkers revealed an increase in bone resorption at 7 and 14 days post-injury, with no change in bone formation at any time points.

**Conclusions:**

High-dose ALN treatment was able to prevent early trabecular bone loss and cartilage degeneration following non-invasive knee injury, but was not able to mitigate long-term joint degeneration. These data contribute to understanding the effect of bisphosphonates on the development of osteoarthritis, and may support the use of anti-resorptive drugs to prevent joint degeneration following injury, although further investigation is warranted.

## Introduction

Approximately 12% of symptomatic osteoarthritis (OA) cases are considered post-traumatic OA (PTOA), corresponding to 5.6 million individuals in the United States being affected by symptomatic PTOA [1]. However, traumatic joint injuries provide a unique opportunity to employ treatments aimed at slowing or preventing the onset of PTOA, as there is an identifiable event that initiates this process. Treatments aimed at inhibiting bone turnover are of particular interest, as subchondral bone is severely affected in OA [2-6], and subchondral bone changes can often be observed before articular cartilage degeneration [3,7-9]. Controlling the bone remodeling process may also be useful for preventing the formation of osteophytes, which are a common and painful outcome of OA development.

The bisphosphonate alendronate (ALN) is a potentially useful therapeutic option for preventing or slowing the development of PTOA. ALN has shown the ability to retain subchondral and periarticular bone following initiation of OA in multiple animal models [10-16]. Importantly, ALN has also exhibited chondroprotective effects [10,12,13,16], and has exhibited the ability to reduce the formation of osteophytes [10,12,15]. However, there is some inconsistency in the reported benefits of ALN for preventing OA; some studies have found no chondroprotective effect in animals treated with ALN [14], or even a negative effect on articular cartilage [11].

Previous studies investigating the effect of ALN on OA development have important limitations. Many studies have focused on mid- to late-stage OA development, which may miss crucial early time points in which subchondral bone undergoes rapid resorption [17]. Another notable shortcoming in the prior studies is the lack of a non-invasive method of joint injury to initiate PTOA. Prior studies typically used invasive surgical means to initiate PTOA, including anterior cruciate ligament (ACL) transection [10,12,13,16] or medial meniscectomy [12,15]. However, these methods may complicate the outcomes of the study due to unintended effects from the invasive surgical procedures. Surgical injury methods may introduce joint swelling or immune response due to the surgical procedures themselves, rather than from the targeted joint disruption. Disruption of the joint capsule also likely affects the natural response of the joint to injury; this is particularly concerning at early time points following injury.

In the current study we investigated the effect of ALN treatment in mice following non-invasive ACL rupture induced by tibial compression overload. We quantified the effect of ALN on early (7 and 14 days post-injury) and long-term (56 days post-injury) cartilage degeneration, subchondral cortical bone thickening, epiphyseal trabecular bone loss, and osteophyte formation. We hypothesized that ALN treatment would inhibit the loss of epiphyseal trabecular bone at early time points, and would decrease long-term subchondral bone thickening, osteophyte formation, and articular cartilage degeneration. These outcomes would support the use of ALN for inhibiting PTOA following traumatic joint injury, as well as provide crucial information about the role of bone turnover in the development of PTOA.

## Methods

### Animals and experimental groups

A total of 90 C57BL/6N female mice (10 weeks old at the time of injury) were obtained from Harlan Sprague Dawley (Indianapolis, IN, USA). Female mice were used rather than male mice to reduce the added activity due to fighting, and to eliminate the confounding effect of individual housing in the event of isolating an aggressor. Mice were randomized into 18 groups based on treatment duration and dose (Table 1). Mice were sacrificed at 7, 14 or 56 days post-injury, and were treated twice weekly with low-dose ALN (ALN-L; 40 μg/kg/dose), high-dose ALN (ALN-H; 1,000 μg/kg/dose), or vehicle solution (VEH; phosphate-buffered saline (PBS)). The 7- and 14-day time points were chosen to quantify the early trabecular bone loss that we have previously observed within 1 to 2 weeks of knee injury in mice [17,18], while the 56-day time point was used as a terminal end point for fully-developed OA including osteophyte formation and articular cartilage degeneration [17]. Mice were cared for in accordance with the guidelines set by the National Institutes of Health (NIH) on the care and use of laboratory animals. Mice were housed in Tecniplast conventional cages (Tecniplast SPA, Buguggiate, Italy), with Bed-o’ Cob bedding (The Andersons Inc., Maumee, OH, USA), with four mice per cage, 12-hour light/dark cycle, 20°C to 26°C ambient temperature. Mice had *ad libitum* access to food (Harlan irradiated 2918 chow) and autoclaved water, and were provided with Enviro-dri and/or Nestlets for environmental enrichment. Mice were monitored by husbandry staff at least once a day, 7 days a week, with monthly healthcare checks by a veterinarian. All procedures were approved by the institutional Animal Studies Committee at UC Davis.

**Table 1:** **Summary of animal numbers used in this study**

	**Day 7**		**Day 14**		**Day 56**	
	**Sham**	**Injured**	**Sham**	**Injured**	**Sham**	**Injured**
Vehicle	4	6	4	6	4	6
Low-dose alendronate	4	6	4	6	4	6
High-dose alendronate	4	6	4	6	4	6

### Tibial compression-induced knee injury

Mice (n = 54; Table 1) were subjected to non-invasive ACL rupture via tibial compression overload as previously described [17,18]. Knee injury was induced by a single dynamic overload cycle (1 mm/s loading rate) to a target compressive load of 12 N using an electromagnetic materials testing system (ELF 3200, Bose, Eden Prairie, MN, USA). This loading protocol causes ACL rupture with associated avulsion fracture from the distal femur [17,18]. Knee injury was noted by a release of compressive force during loading and an audible click. Sham injury was performed by anesthetizing mice (n = 36; Table 1) and applying a 1-2 N compressive load to the lower leg.

### Alendronate treatment

Sixty mice were treated with subcutaneous injection of alendronate sodium trihydrate (Sigma-Aldrich, St Louis, MO, USA) twice weekly (1 injection every 3 to 4 days), starting immediately after injury and lasting until sacrifice at 7, 14 or 56 days. ALN-L mice (n = 30) received doses of 40 μg/kg/dose, while ALN-H mice (n = 30) received doses of 1,000 μg/kg/dose. The low-dose and high-dose treatments were chosen to represent the high and low range of effective doses from previous studies [10,19]. VEH mice (n = 30) were injected twice weekly with a vehicle solution (PBS).

### ELISA analysis of serum biomarkers of bone turnover

Serum biomarkers of bone formation and bone resorption were analyzed at each time point to determine the effect of joint injury and ALN treatment on bone turnover. Blood was collected (100 to 200 μL) from each mouse immediately prior to sacrifice; samples were allowed to clot for 1 to 2 hours, then serum and hematocrit were separated using centrifugation at 5,500 RPM at 4°C. Blood serum was analyzed for cross-linked C-terminal telopeptide of type I collagen (CTX-I) and procollagen type 1 N-terminal propeptide (P1NP) (CUSABio ELISA kit, Wuhan Huamai Biotech Co., Wuhan, China). P1NP is a common serum biomarker of bone formation, while CTX-I is a common serum biomarker of bone resorption [20].

### Micro-computed tomography imaging

Whole joints were scanned postmortem using micro-computed tomography (μCT 35, SCANCO, Brüttisellen, Switzerland) according to the guidelines for μCT analysis of rodent bone structure [21]: energy 55 kVp, intensity 114 mA, integration time 900 ms, 10 μm nominal voxel size. Trabecular bone structure was assessed at the distal femoral epiphysis. The volume of interest for trabecular bone included all trabecular bone enclosed by the growth plate. Trabecular regions were designated on each two-dimensional transverse slice using manually drawn contours that excluded the cortical shell and growth plate. Trabecular bone volume fraction (BV/TV), trabecular thickness (Tb.Th), tissue bone mineral density (BMD; mg HA/cm^3^), and other trabecular bone parameters were directly measured using the manufacturer’s analysis tools. Subchondral cortical bone was assessed at the femoral condyles. The cortical volume of interest included subchondral bone starting at the distal boundary of the femur, extending up to the distal boundary of the growth plate, excluding trabecular bone. Bone volume, cortical thickness (C.Th), and tissue BMD (mg HA/cm^3^) were directly measured using manufacturer’s analysis tools. Osteophyte volume, including all mineralized tissue in and around the joint space, excluding native mineralized structures such as the patella, menisci, and fabella, was measured using a separate contour and was performed on 56-day samples only (based on our previous studies, mineralized osteophytes detectable by μCT are not present to a significant degree at 7 or 14 days post-injury). Systemic effects due to ALN treatment alone (not considering adaptation to injury) were assessed in knees from sham mice.

### Assessment of articular cartilage via whole-joint histology

Following μCT analysis, whole-joint histology was performed on all samples to quantify articular cartilage and joint degeneration. Knees were decalcified for 3 days in 15% formic acid and processed for standard paraffin embedding. Sagittal 6 μm sections were cut across the medial aspect of the joint, separated by 250 μm (four sections for each joint), then stained with Safranin-O and Fast Green. We performed histological analysis only on the medial aspect of the joint because this is the primary site of degeneration for this injury model and similar animal models [17,18,22-25]. For all sections, the articular surfaces of the tibia and femur were graded; blinded slides were graded independently by three readers using the Osteoarthritis Research Society International (OARSI) scale [26]. Grades from the three readers were averaged for each section, then scores for all gradable sections were averaged for each mouse, such that each mouse had one grade for tibial cartilage and one grade for femoral cartilage.

### Statistical analysis

Statistical analysis included a two-tailed paired *t*-test to compare injured limbs to contralateral limbs (the paired *t*-test was used for μCT analysis only). Between-group comparisons for all analyses were performed within each time point using two-way analysis of variance (ANOVA) stratified by injury status and treatment, with *post hoc* analysis using Tukey’s highest significant difference (HSD) test. Significance was set at *P* <0.05 for all tests.

## Results

### Trabecular bone analysis via micro-computed tomography imaging

**Figure 1:**
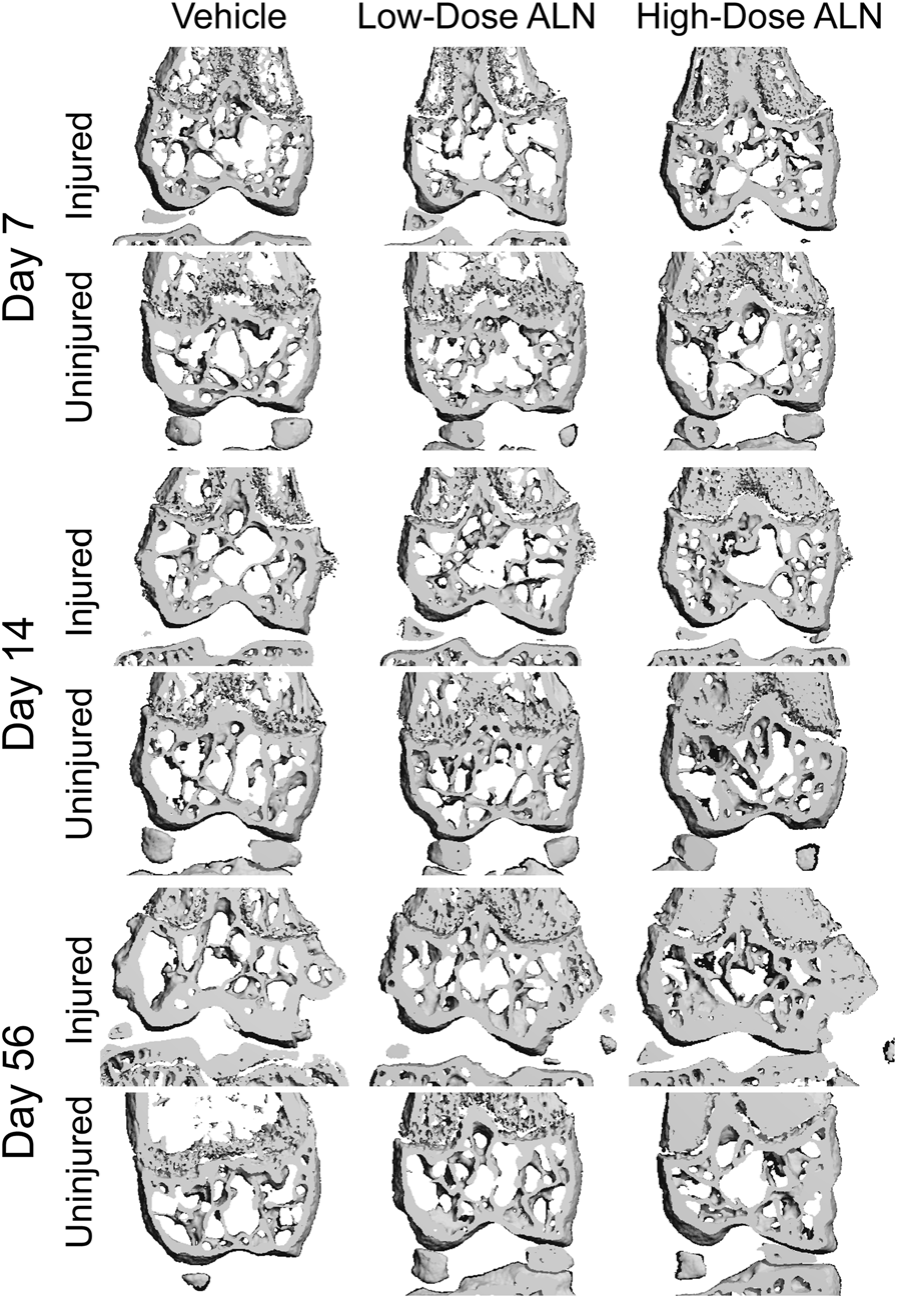
**Representative frontal plane cross-sectional images of the distal femoral epiphysis of injured and sham knees from each of the treatment groups (from micro-computed tomography reconstructions).** Knee injury initiated trabecular bone loss in the femoral epiphysis by 7 days post-injury, but this was largely mitigated by high-dose alendronate (ALN) treatment. Injured knees also exhibited increased subchondral bone thickness by 56 days post-injury, but this was also partially blocked by high-dose ALN treatment. ALN treatment also increased mineralized tissue volume in the metaphysis, likely due to decreased resorption of growth plate cartilage.

**Figure 2:**
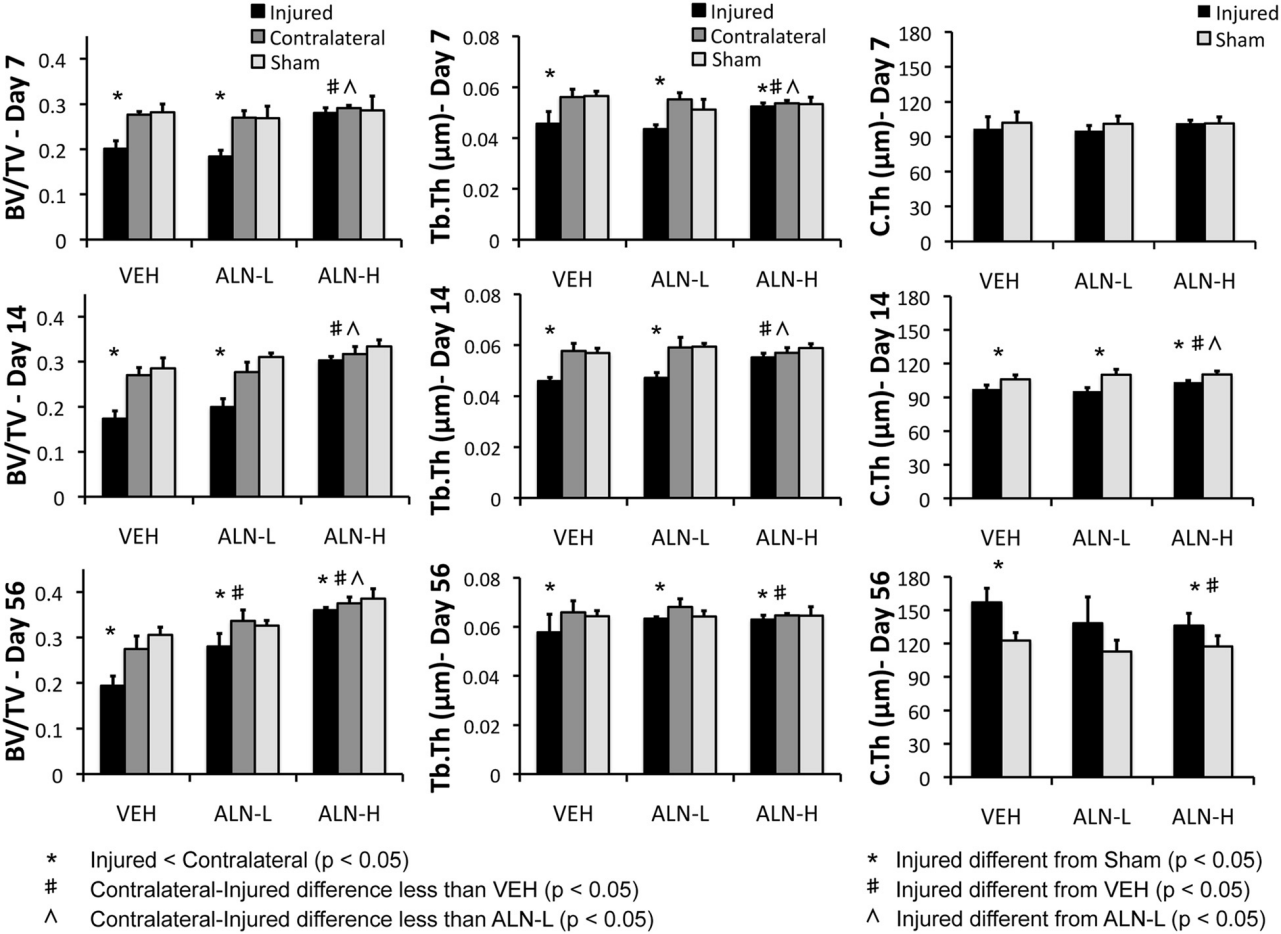
**Epiphyseal trabecular bone volume fraction (BV/TV; left column), trabecular thickness (Tb.Th; center column), and subchondral cortical bone thickness (C.Th; right column) at the distal femur at each time point.** High-dose alendronate (ALN-H) treatment was able to preserve trabecular bone volume and trabecular thickness at all time points, and inhibit sclerosis of the subchondral cortical bone at day 56. ALN-L, low-dose alendronate; VEH, vehicle.

Knee injury induced significant losses of trabecular bone in VEH and ALN-L-treated mice by 7 days post-injury, and persisting until 56 days post-injury, but this effect was largely blocked in ALN-H mice (Figures 1 and 2). For example, VEH and ALN-L mice exhibited 27% and 32% lower BV/TV, respectively, in injured versus uninjured knees at 7 days post-injury, compared to only 4% difference in ALN-H mice. At 56 days post-injury, the injured versus uninjured BV/TV differences were 29%, 16% and 4% for VEH, ALN-L and ALN-H mice, respectively. Trabecular thickness exhibited a similar trend to BV/TV, with noticeable trabecular thinning in the VEH and ALN-L groups, with this effect largely prevented in the ALN-H group. At 7 days post-injury, VEH and ALN-L mice lost 18% and 22% trabecular thickness in the injured knee, respectively, compared to only 2% difference in the ALN-H group. By 56 days post-injury, we observed noticeably smaller differences in Tb.Th between injured and uninjured joints, of 12%, 7% and 3% for the VEH, ALN-L and ALN-H treatments, respectively.

At 7 and 14 days post-injury, ALN-H mice exhibited significantly less trabecular bone loss than VEH or ALN-L mice for BV/TV and Tb.Th, and VEH and ALN-L mice generally showed no significant differences from each other. After 56 days of treatment, however, both ALN-H and ALN-L mice exhibited attenuated trabecular bone loss compared to VEH mice, although ALN-H was notably more effective at preserving trabecular bone volume. BMD of trabecular bone showed no significant differences between any groups for any time points.

Treatment of mice with high- and low-dose ALN also resulted in systemic increases in trabecular bone volume in sham mice. At 7 and 14 days post-injury ALN-H mice exhibited 8% to 14% higher BV/TV values than VEH and ALN-L mice, while ALN-L mice exhibited 2.3% to 2.5% higher values than VEH mice (*P* <0.05). Fifty-six days of ALN-H treatment resulted in a significant increase from ALN-L in BV/TV (11.5%) as well as a 22% increase in BV/TV from VEH to ALN-L. No significant increases were observed for Tb.Th, and no significant differences were observed for BMD at any time points.

### Quantification of subchondral cortical bone

Subchondral cortical bone of injured knees exhibited reduced cortical thickness relative to sham knees at early time points (14 days post-injury in particular), followed by significant subchondral bone thickening by 56 days post-injury (Figures 1 and 2). At 14 days post-injury, VEH and ALN-L cortical thickness was 8% and 13% lower in injured knees than sham knees, respectively, while ALN-H cortical thickness was only 6% lower than sham. In contrast, at 56 days post-injury, VEH and ALN-L cortical thickness was 28% and 23% greater in injured knees than sham knees, respectively, while ALN-H cortical thickness was only 16% greater than sham. No significant differences were observed for bone volume or BMD.

### Quantification of osteophyte volume

**Figure 3:**
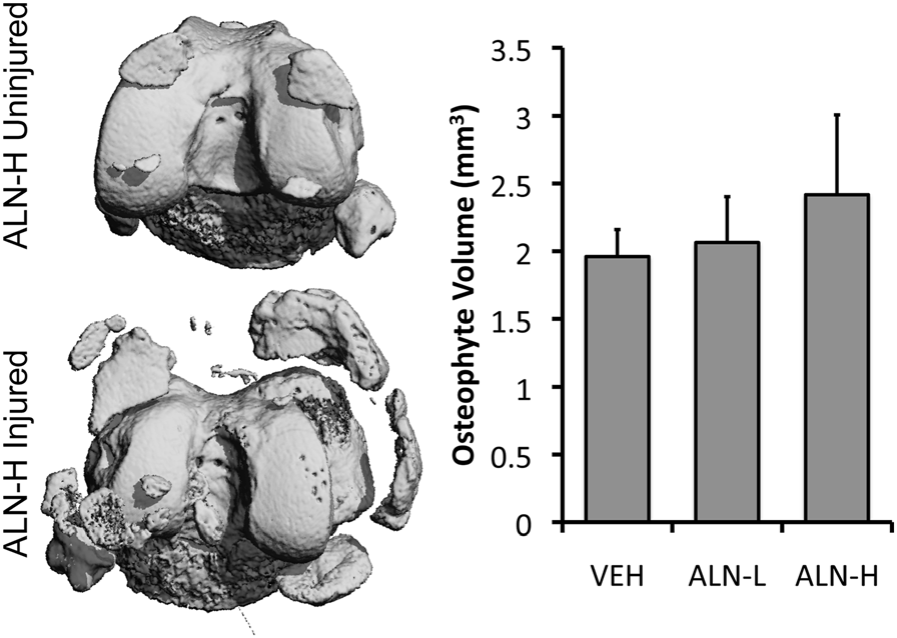
**Injured knees all exhibited considerable osteophyte formation at 56 days post-injury.** Left: inferior images of the distal femur showing the typical pattern of osteophytes. New bone formation occurred primarily from the anterior-medial femur, the posterior-medial tibia, and the medial meniscus. Right: alendronate (ALN) treatment was not effective at preventing osteophyte formation in injured knees. In fact, there was a trend toward increased osteophyte formation in high-dose ALN (ALN-H)-treated animals, although this increase was not statistically significant. ALN-L, low-dose alendronate; VEH, vehicle.

At 56 days post-injury, injured knees exhibited considerable osteophyte formation for all treatment groups (Figure 3). The pattern of osteophyte formation was similar to what we have observed in previous studies [17,18], with osteophytes primarily forming from the anterior-medial femur and the posterior-medial tibia, and with considerable hypertrophy of the medial meniscus. No statistically significant differences in osteophyte volume were observed between VEH, ALN-L or ALN-H mice. However, a trend for increasing osteophyte volume was observed with increased ALN dosage, with nearly 20% increase in osteophyte volume in ALN-H knees compared to VEH knees (*p* = 0.12).

### Whole-joint histology analysis of articular cartilage and joint degeneration

**Figure 4:**
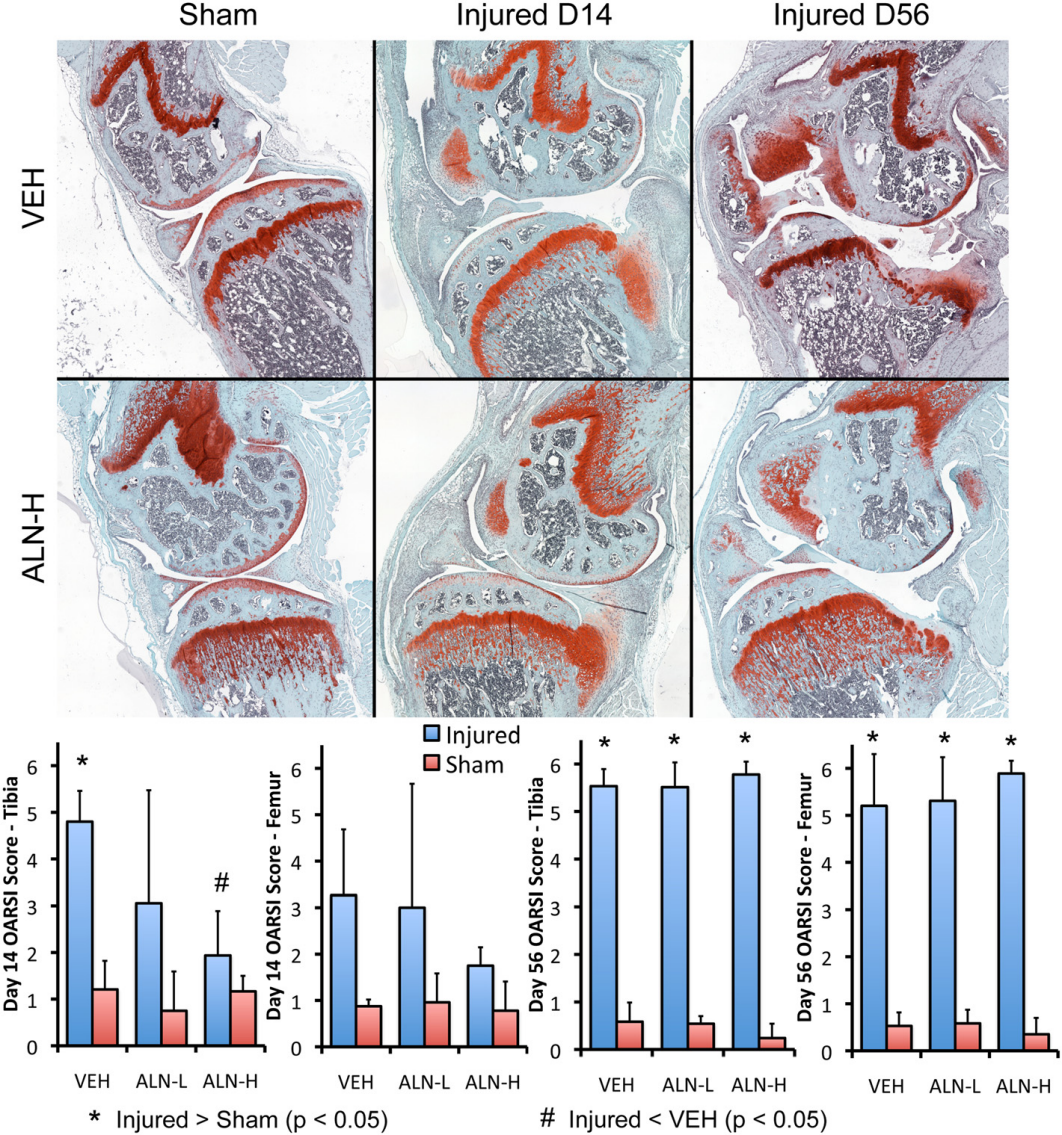
**Representative sagittal plane histological images of the medial aspect of injured and uninjured knees from vehicle (VEH) and high-dose alendronate (ALN-H) groups at 14 and 56 days post-injury.** ALN-H treatment was able to partially preserve articular cartilage at 14 days post-injury, but was not able to affect joint degeneration at day 56. By 7 to 14 days after injury, fibrous formations are evident on the anterior-medial femur and posterior-medial tibia in injured joints, with additional fibrous tissue formation from the menisci. By 56 days post-injury, these fibrous formations have mineralized into osteophytes, and articular cartilage is completely worn away from the posterior surface of the tibia and the articulating surface of the femur. ALN-L, low-dose alendronate; OARSI, Osteoarthritis Research Society International.

Non-invasive knee injury initiated considerable erosion of articular cartilage and joint degeneration in the medial compartment of injured knees from all treatment groups (Figure 4). At 7 days post-injury, only minor cartilage degeneration was apparent in injured knees (OARSI scores of 0 to 3), and no differences were observed between any experimental groups (data not shown). However, moderate articular cartilage degeneration was apparent in injured joints by 14 days post-injury, and joint degeneration was severe in all injured mice by 56 days post-injury. At 14 days post-injury, the tibial surface of ALN-H joints exhibited significantly lower OARSI scores than VEH joints (1.9 ± 0.9 for ALN-H versus 4.8 ± 0.7 for VEH). A similar trend was observed on the femoral surface at day 14 as well, although this difference was not statistically significant. By 7 and 14 days post-injury, injured joints commonly exhibited considerable fibrous or cartilaginous formation at the margins of the joint, particularly from the anterior femur, posterior tibia, and menisci. By 56 days post-injury, all injured joints had developed severe OA (OARSI scores of 5+), and no differences were observed between experimental groups. The pattern of degeneration was similar to what we have observed previously in 12- and 16-week post-injury knees [18], with significant osteophyte formation from the anterior-medial femur, posterior-medial tibia, and hypertrophy of the meniscus, particularly the anterior horn. The anterior portion of articular cartilage of the tibia did not appear to be damaged, most likely due to posterior translation of the distal femur relative to the tibial plateau because of joint instability caused by ACL rupture [18]. The posterior portion of the tibia was severely worn away, with degeneration often extending as far as the growth plate. Lipping was observed at the posterior tibia, similar to that observed in dogs after cranial cruciate ligament rupture [27], with new bone formation occurring from the posterior aspect of the tibia to increase surface area for the articulation between the femur and tibia. Articular cartilage of the femur was also worn away, often reaching the subchondral bone. For the ALN-treated groups, particularly the ALN-H-treated group, unresorbed cartilage from the growth plate was present in the metaphysis of both the tibia and femur, likely due to the absence of osteoclast function.

### Serum biomarker analysis

**Figure 5:**
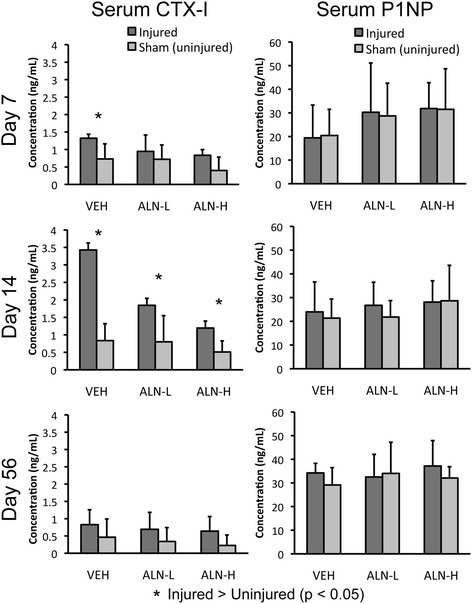
**Serum biomarkers of bone resorption, C-terminal telopeptide of type I collagen (CTX-I) and of bone formation, procollagen type 1 N-terminal propeptide (P1NP) indicated that structural bone changes observed following injury are due to increased bone resorption with no change in bone formation.** CTX-I exhibited the greatest increase in concentration at 14 days post-injury. This increase was effectively reduced by alendronate treatment, but remained higher in injured animals than uninjured animals. No significant differences in P1NP were observed for any experimental groups at any time points. VEH, vehicle; ALN-H, high-dose alendronate; ALN-L, low-dose alendronate.

Injured mice exhibited increased levels of serum CTX-I at all time points compared to uninjured mice for all treatment groups, although this difference was statistically significant primarily at 14 days post-injury (Figure 5). ALN-H treatment resulted in lower sCTX-I values than vehicle at 7 and 14 days (*p* <0.05). sCTX-I was increased 81% in injured VEH mice at day 7, while at day 14 this difference was increased to over 300%. In contrast, ALN-L and ALN-H mice both exhibited approximately 130% increases in sCTX-I at day 14. Serum P1NP (sP1NP) levels were not found to be significantly different between any of the experimental groups at any time points (Figure 5).

## Discussion

In this study, we used a previously characterized model of non-invasive ACL injury in mice to determine both the short-term and long-term effects of ALN on subchondral bone changes and the development of post-traumatic OA. Within 7 to 14 days of injury, we observed a significant loss of trabecular bone in injured knees, consistent with our previous studies. Low-dose ALN treatment was not able to prevent this loss of trabecular bone, whereas high-dose treatment was able to effectively prevent this bone loss. High-dose ALN treatment was also able to inhibit articular cartilage degeneration at 14 days post-injury, although by 56 days post-injury all injured joints exhibited severe OA regardless of treatment. We also observed increased serum levels of CTX-I in injured mice at 7 to 14 days, indicative of a rise in bone resorption, while serum levels of P1NP did not change, indicating no change in bone formation rate. Contrary to previous studies [10,12,13,15,16], osteophyte formation was not affected by ALN use. Altogether, these results confirm the effects of ALN on bone loss, but only partially support the use of antiresorptive agents to protect cartilage and decrease osteophyte formation in OA.

In this study we observed severe joint degeneration in injured knees of all groups of mice at 56 days post-injury, and in fact saw notable degeneration by 14 days post-injury in some mice. This is in contrast to our previous study using this model, in which we observed only minor OA at 56 days post-injury [17]. However, our more recent studies using this model observed a much more severe OA at 12 and 16 weeks post-injury [18] and as early as 56 days post-injury [28]. It is unclear why this more severe degeneration was observed in these recent studies, as the injury methods are identical. This may be partially due to the fact that the current study used female mice rather than male mice as in our previous study [17], although both male and female mice were used by Satkunananthan *et al*. [28], with no observed sex-based differences in OA severity at 56 days. Regardless of the underlying reasons, the results of the current study are consistent with our more recent results [18,28], and with a surgical mouse model of ACL transection [23].

Thickening of subchondral cortical bone is a hallmark symptom of OA. However, a decrease in the structure of underlying (epiphyseal) trabecular bone during OA development is also consistent with clinical observations [3,29,30] and observations from other animal models [22,25]. Consistent with these observations, injured joints in this study exhibited a loss of epiphyseal trabecular bone within 7 days of injury, and a thickening of the subchondral cortical bone by 56 days post-injury, consistent with OA development in humans. Osteophyte growth is another hallmark symptom of OA. In this study we quantified mineralized osteophytes with μCT, defined as all mineralized structures in and around the knee joint, excluding native mineralized structures (patella, fabella, menisci). However, it is important to note that this quantification method only measured mineralized tissue volume, and osteophyte volume obtained using this method is likely to yield different results than those obtained by histology (which would include both mineralized and unmineralized new tissue). Additionally, our analysis did not differentiate between true osteophytes (which are contiguous with the epiphysis of the tibia and femur) and other heterotopic bone formation around the joint (for example, new bone formation contiguous with the meniscus). In this way the osteophyte volume measured by μCT may not be easily translatable to human OA, because mice have a greater propensity to ossify soft tissue structures that are not typically mineralized in humans.

Although high-dose ALN was able to preserve trabecular bone structure, osteophyte formation was not lessened by ALN treatment, and articular cartilage was improved only at an early time point (14 days post-injury). In contrast, Hayami *et al.* observed a chondroprotective and a significant decrease in osteophyte volume with increasing dosage of ALN in rats following ACL transection [10]. Jones *et al*. similarly observed a chondroprotective effect of ALN and reduced osteophyte formation in rats following a surgical knee triad injury [12]. Similar results have been observed following monosodium iodoacetate (MIA) injection in rats [14], medial meniscectomy in rats [15], ACL transection in rabbits [13,16], and in a guinea pig model of OA [11]. The cause of the discrepancies between our results and those from other studies is unclear, but it is possibly due to different animal models or injury methods. Unlike our mouse-based study, these previous studies used larger animal models such as rats or rabbits. These larger animals would have critical differences from mice, including considerably larger body size and thicker articular cartilage. Injury method could also affect the results of these studies. ACL transection or other destabilization methods were used in the majority of these studies, and would therefore produce altered joint biomechanics similar to our mouse model. It is currently unclear how these differences in experimental methods could explain the differences observed in osteophyte and cartilage degeneration. Another possible complicating factor is the fact that our mouse model produced severe OA by 56 days post-injury. It is possible that joint degeneration at this late time point is unavoidable, and is too severe to show significant benefits from therapeutic treatments. However, the significant preservation of articular cartilage with high-dose ALN treatment at 14 days post-injury is supportive of the beneficial effects of antiresorptive therapies on PTOA development. It is possible that the benefits observed at this early time point may be more translatable to OA than the long-term (56 days) observations, as the long-term joint degeneration in this mouse model is much more severe than anything observed in human OA. In this way, the findings from this study may support the use of antiresorptive therapies to prevent or slow OA development.

The efficacy of antiresorptive treatments for preventing OA may depend on several systemic or environmental factors, such as the rate of systemic bone turnover. For example, pamidronate treatment was able to inhibit bone resorption and reduce OA development after medial meniscectomy in ovariectomized mice, in which bone resorption is dramatically increased [31]. Similarly, pamidronate decreased OA development following partial medial meniscectomy in mice overexpressing runt-related transcription factor 2 (Runx2) under the control of collagen type I, which display a high bone remodeling phenotype [32]. These findings support the hypothesis that the rate of bone resorption influences cartilage metabolism and that inhibition might prevent the progression of OA. The current study used young wild-type mice, which do not have an accelerated bone resorption phenotype. This may have affected the efficacy of ALN treatment in this study, which may be more beneficial in a background of high bone turnover. The stage of OA development at which therapy is applied may be another crucial factor determining the efficacy of antiresorptive therapies. For example, immediate and early (4 weeks post-medial-meniscus tear) treatment of rats with zolendronic acid (ZOL) significantly improved subchondral bone microstructure, attenuated cartilage degeneration, reduced weight-bearing asymmetry and calcitonin gene-related peptide (CGRP) expression, while late (8 weeks post-injury) ZOL administration had no significant effects [33]. These data support the idea of a window of opportunity for antiresorptive treatments, which may need to be applied during or before the period of accelerated bone resorption following injury. In the current study, ALN treatment was applied immediately after injury, which was able to preserve trabecular bone mass and partially protect articular cartilage in the ALN-H group. The time frame for effective ALN treatment following injury remains to be determined.

Our analysis of serum bone metabolism biomarkers showed an early rise in a biomarker of bone resorption (sCTX-I) followed by a decline at 56 days post-injury, while a biomarker of bone formation (sP1NP) remained unchanged throughout the study period. This indicates that the early bone changes observed within 14 days of injury are primarily due to increased bone resorption, rather than changes in bone formation. The decreases we observed in sCTX-I with ALN treatment confirm the ability of ALN to diminish bone resorption. Our observations corroborate prior studies showing a decrease in urinary levels of C-terminal telopeptide of type II collagen (CTX-II) and N-terminal telopeptide of type I collagen (NTX-I) with higher dosage of risedronate over time [34,35]. A clinical study by Nishii *et al.* using ALN to treat hip OA in humans also showed a decrease in urinary NTX-I and CTX-II [36]. It is unclear why we did not observe a significant increase in P1NP, even at later time points when there is a strong anabolic response and osteophyte formation. It is possible that the local bone formation response is not strong enough to affect the systemic levels of P1NP that can be measured by serum analysis.

Several clinical studies have investigated the effects of antiresorptive therapies on OA symptoms in human subjects. A study by Carbone *et al.* found that ALN use in OA patients decreased bone abnormalities and attenuated knee pain, yet cartilage degeneration was still present in the magnetic resonance imaging scans of treated patients [37]. Spector *et al.* determined that risedronate use led to significant improvements in the Western Ontario and McMaster Osteoarthritis (WOMAC) index and preservation of knee joint space compared to placebo in a one-year randomized controlled trial involving patients with moderate OA [34]. However, a 2-year randomized controlled trial of risedronate treatment revealed contradictory results, with no significant improvement of WOMAC score or joint space retention in the knee [38]. Similarly, Nishii *et al.* observed no inhibition of OA progression in patients with treated hip OA after 2 years of ALN treatment [36]. Therefore, in spite of the growing body of clinical work investigating the subject, no definitive conclusion can be reached on the practicality of using bisphosphonates to treat patients with OA. Importantly, all of these studies investigated the use of bisphosphonates for treating patients who already had symptomatic OA. We are not aware of any studies that have used antiresorptive therapies as a preventative treatment for slowing the progression of OA following a traumatic joint injury. Our data suggest that ALN may not be effective for preventing the onset of PTOA following joint injury, but this is in contrast to other studies that have shown a chondroprotective effect of ALN, with a decrease in the formation of osteophytes. It is also important to note that in the current study we observed unresorbed cartilage from the growth plate in the metaphysis of mice treated with ALN. This observation is likely not relevant to skeletally mature humans with closed physes, but could be an important concern for using antiresorptive treatments in juvenile subjects.

The mouse model used in this study is somewhat limited because it produces very severe OA by 56 days post-injury, with much more severe joint degeneration than is observed in humans. This is a universal limitation for all mouse models of OA that involve destabilization of the knee. The tissues of the mouse knee are considerably smaller than those of humans or larger animals, and degeneration of the affected joint occurs much faster and to a much greater extent. However, mouse models of OA are crucially important for investigating genetic factors that may increase or decrease the rate of OA development, and for investigating potential therapies, many of which have shown beneficial effects. In this context, we feel that this study is a substantial contribution to the field, because it uses a non-invasive mechanically induced injury to initiate OA. Additionally, we have included investigation of early time points (7 and 14 days post-injury) in order to quantify early or intermediate joint degeneration. The time points of the current study were chosen in order to investigate both early subchondral bone loss and late-stage OA development. However, this excludes many time points between days 14 and 56, during which key events in the development of OA may occur. In spite of this, we were still able to examine the effect of ALN on changes in bone that occur both early and late during PTOA. This study is also somewhat limited because we examined terminal time points at 7, 14, and 56 days post-injury, rather than comparing longitudinal data from the same mice. With an imaging modality such as *in vivo* μCT, longitudinal changes could be directly measured (rather than inferred from cross-sectional data) and the sample size could be reduced. Nonetheless, using cross-sectional data at multiple time points, we were still able to confidently determine the degree to which bone changes occurred in mice post-injury. Finally, the current study used two dosages of ALN, with the high dose having a 25-fold larger concentration than the low dose. The absence of an intermediate dose limited our study to the extent that our data do not inform any conclusions about an optimal dose for OA therapy, or a threshold at which ALN is effective at maintaining subchondral bone structure. However, this limitation did not prevent us from determining with certainty the effect of high- or low-dose ALN on bone metabolism, osteophyte growth, and cartilage damage in our mouse model of knee injury.

Our study had several key strengths that were able to address the limitations of previous studies, including the injury model used and the time points investigated. In this study we used a non-invasive and reproducible ACL rupture model in mice that closely mimics ACL injury in humans. Other studies investigating the effect of bisphosphonates on PTOA have used invasive or non-physiologic injury methods such as ACL transection, medial meniscectomy and MIA injections. By using a non-invasive injury model, we were able to investigate bone changes as early as 7 days after injury, which we have previously shown to be a crucial time for trabecular bone changes in mice during the development of PTOA [17]. Previous studies typically began examining structural changes 2 weeks following initiation of OA [13,14], which may miss critical early time points during which there is rapid turnover of subchondral bone.

## Conclusion

Using a clinically relevant ACL rupture model, we induced PTOA in mice and analyzed the effect of ALN on short-term and long-term bone and cartilage degeneration. High-dose ALN treatment was able to prevent early trabecular bone loss and articular cartilage degeneration following non-invasive joint injury. However, ALN was not able to inhibit osteophyte formation, nor was it able to affect long-term articular cartilage loss or joint degeneration. Our results also suggest that subchondral bone changes initiated by joint injury are primarily due to increased bone resorption, with little change in bone formation at any of the time points quantified. These data contribute to understanding of the effect of bisphosphonates on the development of OA, and may support the use of antiresorptive therapies for slowing or preventing the onset of PTOA following injury. However, more research is necessary to confirm the beneficial effects of antiresorptive therapies for preserving joint health, and to determine the window of opportunity for these treatments.

## Abbreviations

ACL: anterior cruciate ligament
ALN: alendronate
ALN-H: high-dose alendronate (1,000 μg/kg/dose)
ALN-L: low-dose alendronate (40 μg/kg/dose)
BMD: bone mineral density
BV/TV: trabecular bone volume fraction
C.Th: cortical thickness
CGRP: calcitonin gene-related peptide
CTX-I: C-terminal telopeptide of type I collagen
CTX-II: C-terminal telopeptide of type II collagen
ELISA: enzyme-linked immunosorbent assay
MIA: monosodium iodoacetate
NTX-I: N-terminal telopeptide of type I collagen
OA: osteoarthritis
OARSI: Osteoarthritis Research Society International
P1NP: procollagen type 1 N-terminal propeptide
PBS: phosphate-buffered saline
PTOA: post-traumatic osteoarthritis
Runx2: runt-related transcription factor 2
sCTX-I: serum C-terminal telopeptide of type I collagen
sP1NP: serum procollagen type 1 N-terminal propeptide
Tb.Th: trabecular thickness
VEH: vehicle (1 x phosphate-buffered saline)
WOMAC: Western Ontario and McMaster Osteoarthritis index
ZOL: zolendronic acid
μCT: micro-computed tomography
